# Application of single-cell RNA sequencing in preeclampsia

**DOI:** 10.3389/fgwh.2026.1834711

**Published:** 2026-06-11

**Authors:** Xiaojing Pan, Ting Luo

**Affiliations:** Department of Obstetrics, Affiliated Wenling Maternal and Child Health Care Hospital, Wenling City, Zhejiang Province, China

**Keywords:** immune microenvironment, maternal-fetal interface, placenta, preeclampsia, single-cell RNA sequencing, trophoblast

## Abstract

Preeclampsia (PE) is a pregnancy-specific multisystem disorder and a leading cause of maternal and perinatal morbidity and mortality worldwide. Despite the widely accepted two-stage model involving placental dysfunction and maternal systemic inflammation, the precise cellular and molecular mechanisms underlying its pathogenesis remain incompletely understood. In recent years, single-cell RNA sequencing (scRNA-seq) has emerged as a transformative technology capable of resolving transcriptional heterogeneity at unprecedented resolution, offering new insights into the complex cellular landscape of the maternal-fetal interface. This review systematically summarizes the application of scRNA-seq in advancing the understanding of PE pathogenesis. We first introduce the technical principles and advantages of scRNA-seq over bulk sequencing methods. Subsequently, we highlight key findings from scRNA-seq studies of the normal placenta and decidua, establishing a reference for cellular composition and trophoblast differentiation trajectories. We then focus on studies of PE placentas, which have revealed distinct dysfunction in trophoblast subpopulations—including impaired differentiation, invasion, and accelerated senescence—and have identified novel regulatory molecules such as BHLHE40, NDRG1, and DAB2. Additionally, we discuss scRNA-seq-derived insights into immune dysregulation at the maternal-fetal interface, including altered NK cell subsets, macrophage polarization, and disruption of immune tolerance mediated by molecules such as HLA-F and JUNB. Finally, we explore the translational potential of scRNA-seq in identifying novel biomarkers, constructing predictive models, and enabling disease subtyping for precision medicine. By capturing cell-specific transcriptional changes, scRNA-seq provides a powerful framework for deciphering the complexity of PE and holds promise for improving its prediction, diagnosis, and therapy.

## 1 Introduction

Preeclampsia (PE) is a multisystem syndrome characterized by the onset of hypertension and proteinuria after 20 weeks of gestation (1–3). It can affect maternal organs such as the liver, kidneys, and central nervous system, and lead to fetal growth restriction (FGR) (1–3). PE threatens approximately 2%–8% of pregnancies and is a leading cause of maternal and fetal morbidity and mortality. The etiology and pathogenesis of PE are highly complex, involving a combination of genetic, immunological, vascular, and environmental factors (4, 5). It is now widely accepted that the placenta, a unique organ connecting the mother and fetus, plays a pivotal role in the pathogenesis of PE. Insufficient invasion of placental trophoblasts and inadequate remodeling of the uterine spiral arteries in early pregnancy lead to placental ischemia and hypoxia. This, in turn, triggers the release of various anti-angiogenic factors and inflammatory mediators into the maternal circulation, activating a systemic inflammatory response and causing vascular endothelial cell damage, which ultimately manifests as the multisystem clinical features of PE (6–8).

**Table 1:** Key scRNA-Seq studies in preeclampsia and normal placenta.

Study	Sample type	Cell number	Gestational age	Key findings
Tsang et al. (39)	Normal term placenta, early-onset PE placenta	Trophoblast subtypes	Term, early-onset PE	Analyzed >24,000 cells; reconstructed trophoblast differentiation trajectory.
Pavličev et al. (45)	Normal placenta (villous tissue)	Trophoblasts, maternal endometrial stromal cells	Term	Obtained 87 single-cell transcriptomes; investigated cellular interactions between fetal trophoblasts and maternal endometrial stromal cells.
Jiao et al. (46)	PE placenta, gestational diabetes placenta	Trophoblasts, immune cells	Not specified	Identified 96,048 cells into 6 major types (trophoblasts and immune cells). Trophoblasts: 4 subtypes (CTB, VCT, STB, EVT); macrophages: 3 subtypes; lymphocytes: 4 subtypes.
Zhang et al. (47)	PE placenta, healthy control placenta	Villous cytotrophoblast (VCT) subtypes	Not specified (PE vs. control)	First to identify three VCT subtypes: VCT-1 (high expression cluster), VCT-2 (enriched in PE), VCT-3 (mainly in healthy controls, involved in mRNA catabolism).
Zhou et al. (48)	PE placenta	Extravillous trophoblast (EVT) subtypes	Not specified	Found elevated oxidative stress and EVT dysfunction in PE; classified EVTs into four subtypes involved in invasion, immunity, protein synthesis, cell migration, stress response, apoptosis, and oxidative phosphorylation.
Yang et al. (49)	Late-onset PE placenta, control placenta	Trophoblasts, decidual cells	Late-onset PE	Revealed developmental deficiencies in placentation and decidualization in late-onset PE.
Zhang et al. (50)	PE placenta (human, rat)	Trophoblasts	Not specified	BHLHE40 and NDRG1 were overexpressed in PE placentas, accelerating trophoblast senescence; potential key regulatory factors.
Xiao et al. (51)	PE placenta	Villous cytotrophoblasts (VCT), extravillous trophoblasts (EVT)	Not specified	VCT from PE patients tended to differentiate into syncytiotrophoblasts (SCT) rather than EVT; EVT invasive capacity was weakened due to downregulation of TMEM200A.
Wang et al. (52)	PE placenta	Extravillous trophoblasts (EVT)	Not specified	Identified GPER-YAP-Snail-CYR61 signaling axis regulating EVT invasion; CYR61 downregulated in PE placentas, leading to defective invasion.
Liu et al. (53)	Early-onset PE placenta	Extravillous trophoblasts (EVT), decidual vascular smooth muscle cells (dVSMC)	Early-onset PE	DAB2 is a key gene potentially affecting communication between EVT and dVSMC via the CXCL8/PI3K/AKT pathway.
Zheng et al. (54)	Early-onset PE placenta	Syncytiotrophoblasts (SCT), endocrine cells	Early-onset PE	HSD17B1 downregulation impaired SCT differentiation and disrupted estrogen biosynthesis, involved in EOPE pathogenesis.
Huang et al. (55)	PE placenta	Trophoblasts	Not specified	c-Fos expression negatively correlated with neutral lipid accumulation; c-Fos deficiency impaired trophoblast invasion, proliferation, and fusion via p-AMPK/detyrosinated tubulin pathway.
Zadora et al. (56)	PE placenta	Trophoblasts	Not specified	Imprinted gene DLX5 showed loss of imprinting and increased expression in most PE placentas; trophoblasts with high DLX5 exhibited reduced proliferation, increased metabolism, and ER stress.
Hu et al. (57)	Early-onset PE patients, healthy controls	Peripheral blood mononuclear cells (PBMCs)	Early-onset PE	Obtained 80,429 single-cell transcriptomes, identifying 19 cell components in 6 major types. PE patients showed overactivation of B cells, monocytes, NK cells, and suppressed T cell responses.
Whettlock et al. (58)	Uterus (menstrual cycle, early pregnancy)	Uterine NK cells (uNK)	Secretory phase, first trimester	Classified uNK into three functional subsets (uNK1-3); these are most active during implantation and early placentation; dynamic imbalance may be associated with pregnancy complications.
Li et al. (59)	Maternal-fetal interface	Uterine NK cells (uNK), extravillous trophoblasts (EVT)	First trimester	Cytokine signals released by uNK promoted trophoblast differentiation during later invasion stages, regulating gene programs related to blood flow, nutrition, and PE.
Jiang et al. (60)	Early pregnancy decidua	Decidual macrophages	First trimester	Identified three decidual macrophage subsets (CCR2-CD11cLO, CCR2-CD11cHI, CCR2 + CD11cHI) with distinct phagocytic capacities and functions.
Jiang et al. (61)	PE placenta	Macrophages	Not specified	Transcription factor JUNB is a key gene underlying macrophage dysfunction in PE; inhibiting JUNB promotes M2 polarization and enhances trophoblast invasion and angiogenesis via MIIP/PI3K/AKT.
Luo et al. (62)	PE placenta	Trophoblasts, NK cells	Not specified	HLA-F expression was reduced in PE; HLA-F promotes trophoblast proliferation, invasion, migration, and regulates NK cell secretion of immunomodulatory factors, maintaining immune tolerance.
Xiong et al. (63)	Data from PE patients	Not specified (metabolic-based subtyping)	Not specified	Classified PE into distinct subtypes based on metabolic features; screened five key characteristic genes (e.g., RAG1, RBBP7) to build a prediction model.
Botha et al. (64)	PE placenta, maternal blood	Placental cells	Not specified	Placenta-derived CST6/LGMN expression imbalance and elevated maternal blood CST6 levels were associated with PE.
Sun et al. (65)	PE placenta, maternal blood	Trophoblasts	Not specified	TPBG may promote PE by affecting spiral artery remodeling; its level in maternal blood holds predictive potential.
Guo et al. (66)	Early-onset PE, late-onset PE placenta	Placental tissue (bulk transcriptome)	Early-onset PE, late-onset PE	Distinguished molecular features between EOPE and LOPE; identified novel potential biomarkers such as EBI3 and IGF2.
Campbell et al. (67)	Placenta (normal, PE)	Multiple cell types (deconvolution analysis)	Not specified	Constructed a large-scale single-cell reference atlas; demonstrated that many global gene expression differences in PE are driven by changes in cell type proportions (e.g., increased EVTs, decreased mesenchymal cells).

“Not specified” indicates that the original article did not explicitly report the gestational age or only described it as “PE vs. control”; some studies used published data or bioinformatic analysis without specifying sample origin.

Although this theory is widely accepted, the cellular and molecular mechanisms driving the initial placental dysfunction remain incompletely elucidated. Current research primarily focuses on trophoblasts, immune cells, fibroblasts, decidual stromal cells, vascular endothelial cells, among others (9, 10). Traditional research methods, such as microarrays or bulk RNA sequencing (bulk RNA-seq), only provide average gene expression profiles of tissue samples and cannot distinguish the specific alterations occurring within different cell types (e.g., extravillous trophoblasts, syncytiotrophoblasts, maternal immune cells) (9, 11, 12). Single-cell RNA sequencing (scRNA-seq) technology overcomes this bottleneck. This technique sequences genetic information at the single-cell level, enabling the isolation, lysis, amplification, and sequencing of a large number of individual cells from a tissue sample. Consequently, it reveals the gene expression of cellular subpopulations in the normal placenta and the alterations in gene expression profiles of specific cell subpopulations, intercellular communication networks, and novel cellular states under the pathological condition of PE, thereby providing an unprecedented level of granularity (13–16).

This review systematically elaborates on how scRNA-seq technology helps deepens our understanding of PE pathogenesis and discusses its translational potential in the diagnosis and treatment of preeclampsia.

## 2 Single-cell RNA sequencing technology

First reported by Tang et al. (17) in 2009, scRNA-seq is a high-throughput technique capable of performing whole-transcriptome analysis at single-cell resolution.

The core strengths of scRNA-seq lie in its high resolution and sensitivity, enabling the revelation of heterogeneity within cell populations and the identification of rare but functionally important cell subsets. However, it is crucial to acknowledge that scRNA-seq requires high-quality, viable single-cell suspensions. For samples that are difficult to dissociate (e.g., frozen tissues or tissues with complex cell matrices), single-nucleus RNA sequencing (snRNA-seq) is often a more appropriate alternative (18–20). In recent years, with significant technological breakthroughs, its cost has decreased substantially, and automation and throughput have greatly improved, making it possible to analyze millions of cells in a single experiment.

The workflow of scRNA-seq primarily includes single-cell isolation and RNA capture, reverse transcription and cDNA amplification, library construction, high-throughput sequencing, and data analysis (15, 21). Among these, single-cell isolation is a critical step with a major impact on subsequent processing. The isolation must be rapid, efficient, and gentle to preserve the native expression profile. Methods for isolating single-cell samples include limiting dilution, micromanipulation, laser capture microdissection (LCM), fluorescence-activated cell sorting (FACS), magnetic-activated cell sorting (MACS), and microfluidic technology (22–25). Currently, platforms based on MACS and microfluidic technology have become the mainstream choices due to their higher efficiency, scale, and precision. The step of reverse transcription and cDNA amplification is crucial as it determines the sensitivity of sequencing. Most current studies employ the poly-T primer method for reverse transcription. Second-strand synthesis during reverse transcription mainly involves two approaches: one is the polyadenylated tailing method, which is fast but may introduce amplification errors, leading to reduced 5′ end coverage; the other is template switching, which can capture more complete full-length transcripts, reducing 3′ bias caused by incomplete reverse transcription, but with relatively lower sensitivity (26, 27). Following the conversion of RNA to first-strand cDNA, the resulting cDNA is amplified via polymerase chain reaction (PCR) or *in vitro* transcription (IVT) (28). Both methods can introduce amplification biases. To overcome amplification-related biases, unique molecular identifiers (UMIs) are introduced during the reverse transcription step to barcode each individual mRNA molecule within a cell. This improves the quantitative nature of scRNA-seq and enhances read accuracy by effectively eliminating PCR amplification bias (29). After generating barcoded single-cell cDNA, high-throughput sequencing platforms (such as DNBseq based on DNA nanoballs) are used for sequencing, yielding single-stranded circular DNA libraries (15). Finally, data analysis proceeds through multiple steps including sequence alignment, cell quality control, gene quantification, data normalization, removal of confounding factors, dimensionality reduction, clustering analysis, and downstream functional interpretatio (30). Despite its power, scRNA-seq is limited by technical factors such as dissociation bias, cell capture efficiency, and gene dropout events, which must be considered when interpreting data.

**Figure 1:**
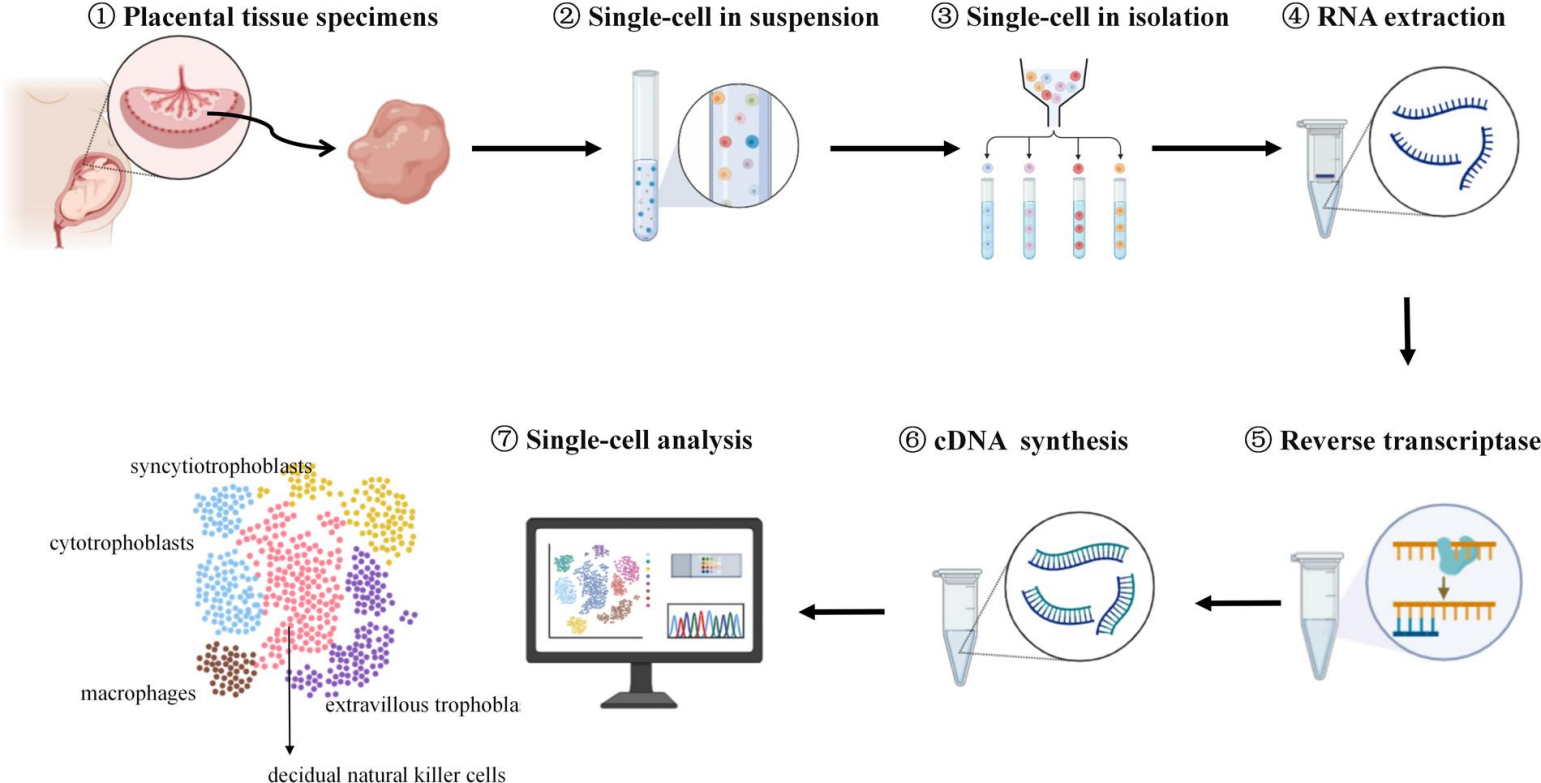
Flowchart of RNA single-cell sequencing.

As a key method for deciphering the interactions between cellular metabolites and gene expression, scRNA-seq has been widely applied in various research fields such as embryonic development, tissue and organ formation, tumor microenvironments, and the immune system, becoming a revolutionary technology driving life sciences research (31–35) (Figure 1).

### 3 Normal placental tissue at single-cell sequencing resolution

The placenta serves as the interface between mother and fetus, responsible for the exchange of gases, nutrients, and waste products, and produces hormones and growth factors that support fetal development and ensure a healthy pregnancy (36). The human placenta consists of a fetal part and a maternal part. The primary functions of the placenta are carried out by trophoblast cells. The term “trophoblast” was first used in 1889 by the Dutch embryologist Ambrosius Arnold Willem Hubrecht (37) to describe cells that transport nutrients and form a protective barrier between mother and fetus. He also observed that trophoblast cells were highly invasive or “erosive.” The placenta has three main types of epithelial trophoblasts: cytotrophoblasts (CTB), syncytiotrophoblasts (STB), and extravillous trophoblasts (EVT) (36, 38). The inner layer CTBs are proliferative cells; the outer layer STBs are the functional cells, differentiated from CTBs (39, 40). EVTs are subdivided into interstitial trophoblasts and endovascular trophoblasts, which cooperate to complete spiral artery remodeling. Interstitial trophoblasts penetrate the decidua, endometrium, and inner third of the myometrium, aggregating around the uterine spiral arteries to prepare for the invasion of endovascular trophoblasts. Endovascular trophoblasts then migrate retrograde along the lumen of the spiral arteries, replacing the vascular endothelium, thereby transforming the narrow, muscular vessels into dilated, low-resistance uteroplacental vessels.

The maternal component of the placenta, the decidua, is composed of decidual immune cells, decidual stromal cells (DSC), and extravillous trophoblasts (EVT). Immune cells include natural killer cells (dNK), macrophages, dendritic cells, T cells, innate lymphoid cells, and B cells, among others, playing a key role in establishing maternal-fetal immune tolerance (41). Single-cell RNA sequencing (scRNA-seq) reveals the presence of at least two distinct fibroblast populations in first-trimester villi, distinguished by the presence or absence of the imprinted gene DLK1 (42, 43). DLK1+ cells exhibit pericyte-like characteristics and may be involved in vasculogenesis (40, 44). These fibroblasts interconnect to form channel networks that eventually converge into the extraembryonic coelom. Within these channels, a type of placental macrophage called Hofbauer cells can be observed, likely playing roles in protecting the fetus from vertical infection, influencing trophoblast and placental vascular development, and transporting nutrients into the extraembryonic coelom (1–3).

Numerous studies have already examined the human placental transcriptome. Pavličev et al. (45) sampled villous tissue from two human term placentas, obtained 87 single-cell transcriptomes, and investigated cellular interactions between fetal trophoblast cells and maternal endometrial stromal cells. Other research performed single-cell transcriptome sequencing on cells from human placentas in the first and second trimesters, identifying novel trophoblast subtypes, Hofbauer cells, and mesenchymal stromal cells (40).

## 4 Placentas from PE pregnancies at single-cell sequencing resolution

During pregnancy, placental gene expression undergoes continuous changes to regulate fetal growth, maintain immune tolerance, and modulate metabolism according to gestational demands. scRNA-seq has been employed to uncover the heterogeneity of different cell types within the human placenta and to elucidate molecular interactions in placental tissues under various conditions.

Jiao et al. (46) analyzed the heterogeneity of human placental cells in preeclampsia (PE) using scRNA-seq. A total of 96,048 cells were identified and categorized into six major cell types, primarily trophoblasts and immune cells. Trophoblasts were further subdivided into four subtypes: cytotrophoblasts (CTB), villous cytotrophoblasts (VCT), syncytiotrophoblasts (STB), and extravillous trophoblasts (EVT). Immune cells were classified into lymphocytes and macrophages. Macrophages comprised three subtypes (decidual macrophages, Hofbauer cells, and macrophages), while lymphocytes included four subtypes (BloodNK, T cells, plasma cells, and decidual natural killer cells).

### 4.1 Trophoblasts

PE is a severe obstetric complication primarily characterized by trophoblast dysfunction and impaired placental development. Trophoblasts are pivotal functional cells in the placenta, playing a crucial role in sustaining normal pregnancy. Significant senescence of trophoblasts leads to placental dysfunction. However, the molecular mechanisms and key regulatory targets governing this process remain poorly understood.

scRNA-seq has advanced the understanding of trophoblasts. Tsang et al. (39) analyzed over 24,000 unlabeled cells from full-term normal pregnancies and early-onset preeclampsia placentas, identifying multiple cellular subtypes within the human placenta and reconstructing the trophoblast differentiation trajectory. Zhang et al. isolated placental tissues from three PE and three healthy pregnant women, obtaining 11,518 cells and further identifying trophoblast subtypes. This study was the first to confirm three subtypes of VCT. VCT-1 exhibited the highest expression cluster; VCT-2 was predominantly distributed in the PE group; VCT-3 was mainly found in the healthy pregnancy group and was involved in nuclear-transcribed mRNA catabolic processes and co-translational protein targeting to membranes (47). Zhou et al. (48) also found elevated oxidative stress and dysfunction in EVTs in PE. Their research identified that EVTs can be divided into four subtypes, concluding that: EVT1 is associated with cell invasion and immunity; EVT2 may be related to protein synthesis and processing, participating in pathways such as ribosome biogenesis; EVT3 is involved in cell migration, chromatin modification, cellular response to stress, etc.; and EVT4 participates in the establishment of endoplasmic reticulum and plasma membrane protein localization, mRNA metabolic processes, intrinsic apoptotic signaling pathway, oxidative phosphorylation, etc.

Comprehensive comparison of cellular subpopulations and transcriptomic differences between PE and normal placentas via scRNA-seq reveals that PE trophoblasts exhibit endoplasmic reticulum stress, abnormalities in specific metabolic pathways, and dysregulation of transcription factor regulatory networks (e.g., modules involving CEBPB, GTF2B) (47–49). Zhang et al. (50) found that BHLHE40 and NDRG1 were both aberrantly overexpressed in PE placentas from both humans and rats, and their overexpression significantly accelerated trophoblast senescence *in vitro*. This suggests BHLHE40 and NDRG1 may be key regulatory factors for trophoblast senescence in PE, holding predictive value for adverse pregnancy outcomes. The differentiation and invasion capabilities of PE trophoblasts are also abnormal. Xiao et al. (51) found that villous cytotrophoblasts (VCT) from PE patients tended to differentiate into syncytiotrophoblasts (SCT) rather than extravillous trophoblasts (EVT). Furthermore, the invasive capacity of EVTs was weakened due to downregulation of the TMEM200A gene. Wang et al. (52) discovered a novel GPER-YAP-Snail-CYR61 signaling axis that regulates EVT invasion. CYR61 expression was significantly downregulated in PE placentas, suggesting this pathway might contribute to defective trophoblast invasion in PE. Liu et al. (53) performed scRNA-seq on whole placental tissue from early-onset preeclampsia (EOPE) patients, identifying DAB2 as a key gene potentially affecting communication between extravillous trophoblasts (EVTs) and decidual vascular smooth muscle cells (dVSMCs) via the CXCL8/PI3K/AKT pathway. PE trophoblasts may also exhibit endocrine abnormalities. Zheng et al. (54) found that HSD17B1 protein levels were downregulated in EOPE patients, leading to impaired SCT differentiation and disrupted estrogen biosynthesis. This indicates HSD17B1 is a dominant coordinator of interactions between trophoblasts and endocrine cells, and impairment of this coordination mechanism may be involved in the pathogenesis of EOPE. Huang et al. (55) found that c-Fos expression levels were negatively correlated with neutral lipid accumulation, and c-Fos deficiency impaired trophoblast invasion, proliferation, and fusion while promoting apoptosis. This change was mediated via the p-AMPK/detyrosinated tubulin pathway. Aberrant gene expression within trophoblasts themselves is also central to PE. Research by Zadora et al. (56) found abnormalities in genomic imprinting in PE placentas. They identified the imprinted gene DLX5, which showed loss of imprinting and increased expression in most PE placentas. *In vitro* experiments demonstrated that trophoblasts with high DLX5 expression exhibited reduced proliferation, increased metabolism, and endoplasmic reticulum stress, with transcriptional profiles resembling PE placentas. Cross-species comparisons indicated DLX5 is a species-specific factor within the regulatory network controlling human trophoblast differentiation. It should be noted that while these *in vitro* and *ex vivo* findings provide strong functional evidence, they do not conclusively establish causality *in vivo*, and the translatability of findings from rodent models requires caution.

### 4.2 Immune cells

A substantial number of immune cells reside at the maternal-fetal interface. Hu et al. isolated peripheral blood mononuclear cells (PBMCs) from three EOPE patients and two healthy controls, obtaining a total of 80,429 single-cell transcriptomes. They identified 19 cellular components belonging to six major cell types: T cells, B cells, NK cells, monocytes, plasmacytoid dendritic cells, and conventional dendritic cells (57).

As pregnancy progresses, individual immune cells exert crucial immunomodulatory and pregnancy-promoting roles through adaptive self-reprogramming throughout gestation. Several studies suggest the occurrence of PE may be associated with functional dysregulation of immune cells and aberrant intercellular communication at the maternal-fetal interface.

NK cells are the most abundant immune cells at the maternal-fetal interface and are vital for regulating trophoblast invasion and vascular remodeling. Whettlock et al. (58) integrated scRNA-seq data to classify uterine NK cells (uNK) into three functionally distinct subsets (uNK1–3). Their study found uNK1 and uNK2 were most active in early pregnancy, and all subsets expressed KIR2D molecules with the highest frequency during the secretory phase and first trimester, enabling interaction with trophoblast HLA-C. This indicates uNK cells are particularly active during the critical periods of implantation and early placentation, and dynamic imbalance among their subsets may be associated with pregnancy complications. Research by Li et al. (59) revealed specific mechanisms by which uNK cells regulate placentation. Using scRNA-seq and organoid models, they found that cytokine signals released by uNK cells promoted trophoblast differentiation during later stages of invasion and regulated gene programs related to blood flow, nutrition, and pregnancy complications like PE.

Decidual macrophages are another key cell type maintaining homeostasis at the maternal-fetal interface, and their dysfunction is closely linked to PE. Jiang et al. (60) identified three decidual macrophage subsets (CCR2-CD11cLO, CCR2-CD11cHI, CCR2 + CD11cHI) with distinct phagocytic capacities and functional properties. Among them, the pro-inflammatory CCR2 + CD11cHI subset and the antioxidant/anti-inflammatory CCR2-CD11cHI subset were primarily located at the trophoblast invasion front, potentially jointly maintaining local inflammatory balance. Further scRNA-seq research by Jiang et al. (61) found the transcription factor JUNB to be a key gene underlying macrophage dysfunction in PE. Inhibiting JUNB promoted macrophage polarization towards the M2 phenotype and, through activation of the MIIP/PI3K/AKT pathway, subsequently enhanced trophoblast invasion and angiogenesis, suggesting targeting JUNB could be a novel strategy for alleviating PE.

The occurrence of PE is related to maternal immune tolerance imbalance towards the embryo. Luo et al. (62) analyzed micro-placentas from PE pregnancies using scRNA-seq, revealing extensive immune disturbances at the maternal-fetal interface. They specifically focused on the non-classical HLA molecule HLA-F, finding its expression was reduced in PE samples. Functional experiments demonstrated that HLA-F expression in trophoblasts promoted their proliferation, invasion, and migration, and regulated NK cell secretion of immunomodulatory factors (e.g., CSF1, CCL22), facilitating the transformation of adaptive NKG2C+ NK cells. This unveils a novel mechanism for HLA-F in maintaining immune tolerance.

The pathology of PE is not confined to the local uterine environment but also involves systemic immune alterations. Hu et al. (57) performed scRNA-seq analysis on PBMCs from PE patients. Their study found that PE patients exhibited overactivation of B cells, monocytes, and NK cells during pregnancy, while immune responses of CD4+ and CD8+ T cells (particularly memory T cells) were suppressed. This imbalanced state could persist postpartum, suggesting its sustained impact on PE pathogenesis and recurrence risk.

scRNA-seq has significantly deepened our understanding of the immune mechanisms in PE. These findings not only partially explain the complex immunological etiology of PE but also provide a crucial basis for developing novel diagnostic biomarkers and therapeutic targets directed at specific immune cell subsets or molecular pathways.

## 5 Conclusion and prospect

scRNA-seq enables detailed analysis of gene expression and can identify molecular dysregulations associated with perturbations or diseases in individual cells, shifting the research paradigm from tissue-level average analysis to cell-specific resolution (40, 43, 44). In recent years, the application of scRNA-seq has revolutionized our understanding of the cellular composition of the placenta (38, 39, 49, 68–70), elucidating the complex cellular and molecular landscape in PE placentas and revealing functional disturbances within trophoblasts as well as in the intricate immune microenvironment at the maternal-fetal interface (13, 71, 72).

scRNA-seq studies have dissected the heterogeneity of the trophoblast lineage, identifying specific subsets of villous cytotrophoblasts and extravillous trophoblasts with disrupted transcriptional programs in preeclampsia. Discoveries include dysregulated differentiation trajectories, impaired invasive capacity associated with specific gene signatures, and the identification of novel regulatory axes (e.g., GPER-YAP-Snail-CYR61, c-Fos/p-AMPK) (53–55). Additionally, trophoblast senescence, endocrine dysfunction, and metabolic stress may contribute to PE development. While many findings have been validated in functional models, suggesting a causal role, these results remain primarily correlative at the transcriptomic level. scRNA-seq has also revealed immune dysregulation in PE patients, including impaired natural killer cell signaling, altered macrophage polarization, disruption of immune tolerance mechanisms, and imbalances such as B/NK cell overactivation coupled with hypoactive T cell responses (59, 61, 62), providing a refined perspective on immune regulation in PE research.

scRNA-seq also facilitates disease subtyping, PE biomarker discovery, and risk prediction model construction. Xiong et al. (63) categorized PE into distinct subtypes based on metabolic features and used machine learning to screen five key characteristic genes, including RAG1 and RBBP7, to build a prediction model. Botha et al. (64) found that placenta-derived CST6/LGMN expression imbalance and elevated maternal blood CST6 levels were associated with PE. Sun et al. (65) proposed that TPBG might promote PE by affecting spiral artery remodeling, and its maternal blood level holds predictive potential. Guo et al. (66) distinguished molecular features between EOPE and LOPE and identified novel potential biomarkers such as EBI3 and IGF2.

scRNA-seq also offers a new perspective for interpreting traditional bulk transcriptome data. Campbell et al. (67) constructed a large-scale placental single-cell reference atlas and, through “deconvolution” analysis, demonstrated that many global gene expression differences in PE are actually mediated by changes in cellular composition proportions. This underscores the importance of considering cellular heterogeneity in research.

scRNA-seq also has limitations, and technical and biological hurdles need to be addressed. First, differences exist between early-onset and late-onset preeclampsia (PE). Placentas from early-onset PE often exhibit more pronounced defects in extravillous trophoblast (EVT) invasion and spiral artery remodeling, as well as stronger signatures of hypoxia and endoplasmic reticulum stress, whereas late-onset PE may involve more metabolic or inflammatory pathways. Studies should make this distinction. Second, multiple independent studies consistently report trophoblast dysfunction and immune dysregulation in PE. However, discrepancies exist in the markers and transcriptomic profiles of specific cell subtypes. For example, the number of trophoblast subtypes and their defining gene signatures vary across studies. These differences likely arise from variations in clinical sample heterogeneity, placental sampling location, gestational age at collection, and bioinformatics analysis pipelines. Furthermore, research in this field faces challenges due to methodological heterogeneity. Differences in tissue dissociation protocols, cell capture platforms, sequencing depth, and data analysis may lead to divergent conclusions. Harmonization efforts and the use of common reference atlases are needed to overcome these obstacles. We also need to address the high cost and complexity of this technology. Moreover, scRNA-seq is not well-suited to capture the multinucleated structure of syncytiotrophoblasts, necessitating the use of other omics approaches to compensate for this limitation (30, 73–76).

This review systematically elaborates on the important role and limitations of scRNA-seq in investigating trophoblast and immune cell functions in preeclamptic placentas. It is hoped that the application of scRNA-seq and ongoing technological advances will accelerate the translation of these fundamental discoveries into clinically actionable strategies for the prediction, prevention, and personalized treatment of PE, ultimately improving maternal and fetal outcomes.

## References

[1] Kaufmann P Stark J Stegner HE. The villous stroma of the human placenta. I. The ultrastructure of fixed connective tissue cells. Cell Tissue Res. (1977) 177(1):105–21. 10.1007/BF00221122 837398

[2] Burton GJ. The fine structure of the human placental villus as revealed by scanning electron microscopy. Scanning Microsc. (1987) 1(4):1811–28. 3324327

[3] Castellucci M Kosanke G Verdenelli F Huppertz B Kaufmann P. Villous sprouting: fundamental mechanisms of human placental development. Hum Reprod Update. (2000) 6(5):485–94. 10.1093/humupd/6.5.485 11045879

[4] Goldman-Wohl D Yagel S. Regulation of trophoblast invasion: from normal implantation to pre-eclampsia. Mol Cell Endocrinol. (2002) 187(1–2):233–8. 10.1016/S0303-7207(01)00687-6 11988332

[5] Brosens I Pijnenborg R Vercruysse L Romero R. The “great obstetrical syndromes” are associated with disorders of deep placentation. Am J Obstet Gynecol. (2011) 204(3):193–201. 10.1016/j.ajog.2010.08.009 21094932 PMC3369813

[6] Egbor M Ansari T Morris N Green CJ Sibbons PD. Maternal medicine: morphometric placental villous and vascular abnormalities in early- and late-onset pre-eclampsia with and without fetal growth restriction. BJOG. (2006) 113(5):580–9. 10.1111/j.1471-0528.2006.00882.x 16579806

[7] Staff AC Redman CWG Williams D Leeson P Moe K Thilaganathan B. Pregnancy and long-term maternal cardiovascular health: progress through harmonization of research cohorts and biobanks. Hypertension. (2016) 67(2):251–60. 10.1161/HYPERTENSIONAHA.115.06357 26667417

[8] Yagel S Cohen SM Goldman-Wohl D. An integrated model of preeclampsia: a multifaceted syndrome of the maternal cardiovascular-placental-fetal array. Am J Obstet Gynecol. (2022) 226(2S):S963–72. 10.1016/j.ajog.2020.10.023 33712272

[9] Li Y Ma L Wu D Chen G. Advances in bulk and single-cell multi-omics approaches for systems biology and precision medicine. Brief Bioinform. (2021) 22(5):bbab024. 10.1093/bib/bbab024 33778867

[10] Shu C Street K Breton CV Bastain TM Wilson ML. A review of single-cell transcriptomics and epigenomics studies in maternal and child health. Epigenomics. (2024) 16(10):775–93. 10.1080/17501911.2024.2343276 38709139 PMC11318716

[11] Kashima Y Sakamoto Y Kaneko K Seki M Suzuki Y Suzuki A. Single-cell sequencing techniques from individual to multiomics analyses. Exp Mol Med. (2020) 52(9):1419–27. 10.1038/s12276-020-00499-2 32929221 PMC8080663

[12] Yuan Q Lv N Chen Q Shen S Wang Y Tong J. Application of single cell sequencing technology in ovarian cancer research (review). Funct Integr Genomics. (2024) 24(5):144. 10.1007/s10142-024-01432-w 39196391 PMC11358195

[13] Haque A Engel J Teichmann SA Lönnberg T. A practical guide to single-cell RNA-Sequencing for biomedical research and clinical applications. Genome Med. (2017) 9(1):75. 10.1186/s13073-017-0467-4 28821273 PMC5561556

[14] Potter SS. Single-cell RNA sequencing for the study of development, physiology and disease. Nat Rev Nephrol. (2018) 14(8):479–92. 10.1038/s41581-018-0021-7 29789704 PMC6070143

[15] Jovic D Liang X Zeng H Lin L Xu F Luo Y. Single-cell RNA sequencing technologies and applications: a brief overview. Clin Transl Med. (2022) 12(3):e694. 10.1002/ctm2.694 35352511 PMC8964935

[16] Walter TJ Suter RK Ayad NG. An overview of human single-cell RNA sequencing studies in neurobiological disease. Neurobiol Dis. (2023) 184:106201. 10.1016/j.nbd.2023.106201 37321420 PMC10470823

[17] Tang F Barbacioru C Wang Y Nordman E Lee C Xu N. mRNA-Seq whole-transcriptome analysis of a single cell. Nat Methods. (2009) 6(5):377–82. 10.1038/nmeth.1315 19349980

[18] Thomsen ER Mich JK Yao Z Hodge RD Doyle AM Jang S. Fixed single-cell transcriptomic characterization of human radial glial diversity. Nat Methods. (2016) 13(1):87–93. 10.1038/nmeth.3629 26524239 PMC4869711

[19] Haber AL Biton M Rogel N Herbst RH Shekhar K Smillie C. A single-cell survey of the small intestinal epithelium. Nature. (2017) 551(7680):333–9. 10.1038/nature24489 29144463 PMC6022292

[20] Denisenko E Guo BB Jones M Hou R De Kock L Lassmann T. Systematic assessment of tissue dissociation and storage biases in single-cell and single-nucleus RNA-seq workflows. Genome Biol. (2020) 21(1):130. 10.1186/s13059-020-02048-6 32487174 PMC7265231

[21] De Klerk E ‘t Hoen PA. Alternative mRNA transcription, processing, and translation: insights from RNA sequencing. Trends Genet. (2015) 31(3):128–39. 10.1016/j.tig.2015.01.001 25648499

[22] Warren L Bryder D Weissman IL Quake SR. Transcription factor profiling in individual hematopoietic progenitors by digital RT-PCR. Proc Natl Acad Sci U S A. (2006) 103(47):17807–12. 10.1073/pnas.0608512103 17098862 PMC1693828

[23] Zhao X-M Cui L-S Hao H-S Wang H-Y Zhao S-J Du W-H. Transcriptome analyses of inner cell mass and trophectoderm cells isolated by magnetic-activated cell sorting from bovine blastocysts using single cell RNA-seq. Reprod Domest Anim. (2016) 51(5):726–35. 10.1111/rda.12737 27440443

[24] Han X Wang R Zhou Y Fei L Sun H Lai S. Mapping the mouse cell atlas by microwell-seq. Cell. (2018) 172(5):1091–1107.e17. 10.1016/j.cell.2018.02.001 29474909

[25] Valihrach L Androvic P Kubista M. Platforms for single-cell collection and analysis. Int J Mol Sci. (2018) 19(3):807. 10.3390/ijms19030807 29534489 PMC5877668

[26] Ramsköld D Luo S Wang Y-C Li R Deng Q Faridani OR. Full-length mRNA-Seq from single-cell levels of RNA and individual circulating tumor cells. Nat Biotechnol. (2012) 30(8):777–82. 10.1038/nbt.2282 22820318 PMC3467340

[27] Sasagawa Y Nikaido I Hayashi T Danno H Uno KD Imai T. Quartz-Seq: a highly reproducible and sensitive single-cell RNA sequencing method, reveals non-genetic gene-expression heterogeneity. Genome Biol. (2013) 14(4):R31. 10.1186/gb-2013-14-4-r31 23594475 PMC4054835

[28] Goetz JJ Trimarchi JM. Transcriptome sequencing of single cells with smart-seq. Nat Biotechnol. (2012) 30(8):763–5. 10.1038/nbt.2325 22871714

[29] Fu GK Hu J Wang PH Fodor SP. Counting individual DNA molecules by the stochastic attachment of diverse labels. Proc Natl Acad Sci U S A. (2011) 108(22):9026–31. 10.1073/pnas.1017621108 21562209 PMC3107322

[30] Rana S Lemoine E Granger JP Karumanchi SA. Preeclampsia: pathophysiology, challenges, and perspectives. Circ Res. (2019) 124(7):1094–112. 10.1161/CIRCRESAHA.118.313276 30920918

[31] Van Galen P Hovestadt V Wadsworth MH II Hughes TK Griffin GK Battaglia S. Single-Cell RNA-Seq reveals AML hierarchies relevant to disease progression and immunity. Cell. (2019) 176(6):1265–1281.e24. 10.1016/j.cell.2019.01.031 30827681 PMC6515904

[32] Liao J Lu X Shao X Zhu L Fan X. Uncovering an organ’s molecular architecture at single-cell resolution by spatially resolved transcriptomics. Trends Biotechnol. (2021) 39(1):43–58. 10.1016/j.tibtech.2020.05.006 32505359

[33] Srivatsan SR Regier MC Barkan E Franks JM Packer JS Grosjean P. Embryo-scale, single-cell spatial transcriptomics. Science. (2021) 373(6550):111–7. 10.1126/science.abb9536 34210887 PMC9118175

[34] Wu F Fan J He Y Xiong A Yu J Li Y. Single-cell profiling of tumor heterogeneity and the microenvironment in advanced non-small cell lung cancer. Nat Commun. (2021) 12(1):2540. 10.1038/s41467-021-22801-0 33953163 PMC8100173

[35] Wang J Zhu N Su X Gao Y Yang R. Novel tumor-associated macrophage populations and subpopulations by single cell RNA sequencing. Front Immunol. (2023) 14:1264774. 10.3389/fimmu.2023.1264774 38347955 PMC10859433

[36] Li H Huang Q Liu Y Garmire LX. Single cell transcriptome research in human placenta. Reproduction. (2020) 160(6):R155–67. 10.1530/REP-20-0231 33112783 PMC7707799

[37] Pijnenborg R Vercruysse LAAW. Hubrecht and the naming of the trophoblast. Placenta. (2013) 34(4):314–9. 10.1016/j.placenta.2013.01.002 23395301

[38] Derisoud E Jiang H Zhao A Chavatte-Palmer P Deng Q. Revealing the molecular landscape of human placenta: a systematic review and meta-analysis of single-cell RNA sequencing studies. Hum Reprod Update. (2024) 30(4):410–41. 10.1093/humupd/dmae006 38478759 PMC11215163

[39] Tsang JCH Vong JSL Ji L Poon LCY Jiang P Lui KO. Integrative single-cell and cell-free plasma RNA transcriptomics elucidates placental cellular dynamics. Proc Natl Acad Sci U S A. (2017) 114(37):E7786–95. 10.1073/pnas.1710470114 28830992 PMC5604038

[40] Liu Y Fan X Wang R Lu X Dang Y-L Wang H. Single-cell RNA-seq reveals the diversity of trophoblast subtypes and patterns of differentiation in the human placenta. Cell Res. (2018) 28(8):819–32. 10.1038/s41422-018-0066-y 30042384 PMC6082907

[41] Yang F Zheng Q Jin L. Dynamic function and composition changes of immune cells during normal and pathological pregnancy at the maternal-fetal interface. Front Immunol. (2019) 10:2317. 10.3389/fimmu.2019.02317 31681264 PMC6813251

[42] Kagami M Matsuoka K Nagai T Yamanaka M Kurosawa K Suzumori N. Paternal uniparental disomy 14 and related disorders: placental gene expression analyses and histological examinations. Epigenetics. (2012) 7(10):1142–50. 10.4161/epi.21937 22917972 PMC3469456

[43] Vento-Tormo R Efremova M Botting RA Turco MY Vento-Tormo M Meyer KB. Single-cell reconstruction of the early maternal-fetal interface in humans. Nature. (2018) 563(7731):347–53. 10.1038/s41586-018-0698-6 30429548 PMC7612850

[44] Suryawanshi H Morozov P Straus A Sahasrabudhe N Max KEA Garzia A. A single-cell survey of the human first-trimester placenta and decidua. Sci Adv. (2018) 4(10):eaau4788. 10.1126/sciadv.aau4788 30402542 PMC6209386

[45] Pavličev M Wagner GP Chavan AR Owens K Maziarz J Dunn-Fletcher C. Single-cell transcriptomics of the human placenta: inferring the cell communication network of the maternal-fetal interface. Genome Res. (2017) 27(3):349–61. 10.1101/gr.207597.116 28174237 PMC5340963

[46] Jiao B Wang Y Li S Lu J Liu J Xia J. Dissecting human placental cells heterogeneity in preeclampsia and gestational diabetes using single-cell sequencing. Mol Immunol. (2023) 161:104–18. 10.1016/j.molimm.2023.07.005 37572508

[47] Zhang T Bian Q Chen Y Wang X Yu S Liu S. Dissecting human trophoblast cell transcriptional heterogeneity in preeclampsia using single-cell RNA sequencing. Mol Genet Genomic Med. (2021) 9(8):e1730. 10.1002/mgg3.1730 34212522 PMC8404237

[48] Zhou W Wang H Yang Y Guo F Yu B Su Z. Trophoblast cell subtypes and dysfunction in the placenta of individuals with preeclampsia revealed by single-cell RNA sequencing. Mol Cells. (2022) 45(5):317–28. 10.14348/molcells.2021.0211 35289305 PMC9095508

[49] Yang J Gong L Liu Q Zhao H Wang Z Li X. Single-cell RNA-seq reveals developmental deficiencies in both the placentation and the decidualization in women with late-onset preeclampsia. Front Immunol. (2023) 14:1142273. 10.3389/fimmu.2023.1142273 37283740 PMC10239844

[50] Zhang Y Chu J Yu Z Gong Y. Single cell RNA-sequencing reveals BHLHE40 and NDRG1 as key regulatory molecules modulating the trophoblastic senescence in preeclampsia. Placenta. (2025) 168:74–87. 10.1016/j.placenta.2025.06.009 40517485

[51] Xiao S Ding Y Yu L Deng Y Zhou Y Peng M. Maternal-Fetal interface cell dysfunction in patients with preeclampsia revealed via single-cell RNA sequencing. Am J Reprod Immunol. (2025) 94(3):e70101. 10.1111/aji.70101 40924867

[52] Wang H Han X Wu Z Guo M Luo S Fang L. G protein-coupled estrogen receptor promotes human extravillous trophoblast invasion via YAP-snail-mediated CYR61 expression. Cell Signal. (2025) 135:112033. 10.1016/j.cellsig.2025.112033 40744331

[53] Liu Y Du L Gu S Liang J Huang M Huang L. Identification of the role of DAB2 and CXCL8 in uterine spiral artery remodeling in early-onset preeclampsia. Cell Mol Life Sci. (2024) 81(1):180. 10.1007/s00018-024-05212-4 38613672 PMC11016014

[54] Zheng S Feng W Sun Z Xu P Dong S Pan L. HSD17B1-mediated Trophoblast differentiation lowers estrogen levels in early-onset preeclampsia. Sci Rep. (2025) 15(1):17448. 10.1038/s41598-025-02490-1 40394177 PMC12092795

[55] Huang Y Zhao S Zeng C Shi S Li Z Shen L. c-Fos mediates preeclampsia through p-AMPK/detyrosinated tubulin pathway. Hypertension. (2025) 82(11):1959–74. 10.1161/HYPERTENSIONAHA.124.24416 40859841

[56] Zadora J Singh M Herse F Przybyl L Haase N Golic M. Disturbed placental imprinting in preeclampsia leads to altered expression of DLX5, a human-specific early trophoblast marker. Circulation. (2017) 136(19):1824–39. 10.1161/CIRCULATIONAHA.117.028110 28904069 PMC5671803

[57] Hu J Guo Q Liu C Yu Q Ren Y Wu Y. Immune cell profiling of preeclamptic pregnant and postpartum women by single-cell RNA sequencing. Int Rev Immunol. (2024) 43(1):1–12. 10.1080/08830185.2022.2144291 36369864

[58] Whettlock EM Woon EV Cuff AO Browne B Johnson MR Male V. Dynamic changes in uterine NK cell subset frequency and function over the menstrual cycle and pregnancy. Front Immunol. (2022) 13:880438. 10.3389/fimmu.2022.880438 35784314 PMC9245422

[59] Li Q Sharkey A Sheridan M Magistrati E Arutyunyan A Huhn O. Human uterine natural killer cells regulate differentiation of extravillous trophoblast early in pregnancy. Cell Stem Cell. (2024) 31(2):181–195.e9. 10.1016/j.stem.2023.12.013 38237587

[60] Jiang X Du MR Li M Wang H. Three macrophage subsets are identified in the uterus during early human pregnancy. Cell Mol Immunol. (2018) 15(12):1027–37. 10.1038/s41423-018-0008-0 29618777 PMC6269440

[61] Jiang P Zhu X Jiang Y Li H Luo Q. Targeting JUNB to modulate M2 macrophage polarization in preeclampsia. Biochim Biophys Acta Mol Basis Dis. (2024) 1870(6):167194. 10.1016/j.bbadis.2024.167194 38663490

[62] Luo F Liu F Guo Y Xu W Li Y Yi J. Single-cell profiling reveals immune disturbances landscape and HLA-F-mediated immune tolerance at the maternal-fetal interface in preeclampsia. Front Immunol. (2023) 14:1234577. 10.3389/fimmu.2023.1234577 37854606 PMC10579943

[63] Xiong Z Guan H Pei S Wang C. Identification of metabolism-related subtypes and feature genes of pre-eclampsia. Sci Rep. (2025) 15(1):4986. 10.1038/s41598-025-89140-8 39930027 PMC11811273

[64] Botha SM Bartho LA Hartmann S Cannon P Nguyen A Nguyen T-V. Cystatin 6 (CST6) and legumain (LGMN) are potential mediators in the pathogenesis of preeclampsia. Sci Rep. (2025) 15(1):12945. 10.1038/s41598-025-96823-9 40234537 PMC12000359

[65] Sun L Shi M Wang J Han X Wei J Huang Z. Overexpressed trophoblast glycoprotein contributes to preeclampsia development by inducing abnormal trophoblast migration and invasion toward the uterine spiral artery. Hypertension. (2024) 81(7):1524–36. 10.1161/HYPERTENSIONAHA.124.22923 38716674

[66] Guo F Zhang B Yang H Fu Y Wang Y Huang J. Systemic transcriptome comparison between early- and late-onset pre-eclampsia shows distinct pathology and novel biomarkers. Cell Prolif. (2021) 54(2):e12968. 10.1111/cpr.12968 33332660 PMC7848957

[67] Campbell KA Colacino JA Puttabyatappa M Dou JF Elkin ER Hammoud SS. Placental cell type deconvolution reveals that cell proportions drive preeclampsia gene expression differences. Commun Biol. (2023) 6(1):264. 10.1038/s42003-023-04623-6 36914823 PMC10011423

[68] Pique-Regi R Romero R Tarca AL Sendler ED Xu Y Garcia-Flores V. Single cell transcriptional signatures of the human placenta in term and preterm parturition. Elife. (2019) 8:e52004. 10.7554/eLife.52004 31829938 PMC6949028

[69] Wang Q Li J Wang S Deng Q An Y Xing Y. Single-cell transcriptional profiling reveals cellular and molecular divergence in human maternal-fetal interface. Sci Rep. (2022) 12(1):10892. 10.1038/s41598-022-14516-z 35764880 PMC9240006

[70] Garcia-Flores V Romero R Tarca AL Peyvandipour A Xu Y Galaz J. Deciphering maternal-fetal cross-talk in the human placenta during parturition using single-cell RNA sequencing. Sci Transl Med. (2024) 16(729):eadh8335. 10.1126/scitranslmed.adh8335 38198568 PMC11238316

[71] Zhang L Li Z Skrzypczynska KM Fang Q Zhang W O’Brien SA. Single-Cell analyses inform mechanisms of myeloid-targeted therapies in colon cancer. Cell. (2020) 181(2):442–459.e29. 10.1016/j.cell.2020.03.048 32302573

[72] Chang X Zheng Y Xu K. Single-Cell RNA sequencing: technological progress and biomedical application in cancer research. Mol Biotechnol. (2024) 66(7):1497–519. 10.1007/s12033-023-00777-0 37322261 PMC11217094

[73] Pique-Regi R Romero R Tarca AL Luca F Xu Y Alazizi A. Does the human placenta express the canonical cell entry mediators for SARS-CoV-2. Elife. (2020) 9:e58716. 10.7554/eLife.58716 32662421 PMC7367681

[74] Arutyunyan A Roberts K Troulé K Wong FCK Sheridan MA Kats I. Spatial multiomics map of trophoblast development in early pregnancy. Nature. (2023) 616(7955):143–51. 10.1038/s41586-023-05869-0 36991123 PMC10076224

[75] Gao L Mathur V Tam SKM Zhou X Cheung MF Chan LY. Single-cell analysis reveals transcriptomic and epigenomic impacts on the maternal-fetal interface following SARS-CoV-2 infection. Nat Cell Biol. (2023) 25(7):1047–60. 10.1038/s41556-023-01169-x 37400500 PMC10344786

[76] Wang M Liu Y Sun R Liu F Li J Yan L. Single-nucleus multi-omic profiling of human placental syncytiotrophoblasts identifies cellular trajectories during pregnancy. Nat Genet. (2024) 56(2):294–305. 10.1038/s41588-023-01647-w 38267607 PMC10864176

